# The Social Importance of Vital Statistics

**Published:** 1917-02

**Authors:** 


**A Monthly Review of Medicine and Surgery**

EDITOR AND PUBLISHER, DR. A. L. BENEDICT, 228 Summer St., corner of Elmwood Ave., Buffalo, (Address for all communications. Please make personal and telephone calls before 1 P. M.)

Third, in age, on the western continent. Independent, but supports professional organizations. News limited mainly to Western N. Y. and Penn.

Subscription $2.00 a year; $3.00 for two years in advance. Changes and errors in address or failure or duplication of delivery, should be reported immediately.

Yearly Volume 72 FEBRUARY, 1917 Number 7

### The Social Importance of Vital Statistics.

It is unnecessary to emphasize the interest and practical value of accurate vital statistics, medically or sociologically. To know the magnitude and exact nature of disease is a long step toward its prevention. Statistics of diseases and death cannot be compiled without reference to population, and hence virtually include the need of other vital statistics, as of birth and marriage.

A generation ago, an excellent family, temporarily in straitened circumstances, placed two little girls in an orphan asylum. They were adopted by two other families and, while leading lives far separated, remained in touch with each other. Their parents, regaining financial independence and even a moderate degree of prosperity, applied for the children but the institution either could not or would not give information as to their whereabouts. The pride and indomitable perseverance of one of the little girls ultimately led to the discovery of the parents, though not to the literal reunion of the family, as family relations with foster parents had been too intimately established and both girls had married.

In the absence of statistics of birth, marriage, etc., kept by local governments, church records or the individual notes of ministers and physicians and undertakers, are often sought, sometimes many years or even generations after the death of the original keeper of the records. Sometimes, the motive is the eminently practical one of establishing a financial claim; sometimes the honor of a woman and of her children is involved as by the establishment of a legal marriage; sometimes the motive is the readily understood seeking of parents or children by those separated by circumstances though near in ties of blood and natural affection. Ridiculous as it may seem, the pathetic yearning for ancestors or for the social distinction of eligibility to be the son or daughter of an organization often seems to outweigh any possible financial considerations. In the support of several special magazines, of several hundred professional genealogists, in postage and for transportation, our people annually spend several millions a year for this purpose, quite aside from dues and incidental expenses in connection with societies which, of course, yield a social and educational return, not to mention the patriotic sentiment involved. And it should be remembered that very little of this expense is incurred for fictious or genuine titles but that information is wanted as to descent from very plain people. However we may deride this demand for old vital statistics, it represents, by the reliable criterion of willingness to spend money, and time, a very real desire.

The medical profession necessarily has an important obligation in regard to vital statistics, both from the social and the medical standpoint. Its members should impress the importance of such records upon the laity and should secure accurate registration in all cases.

